# Veterinarian–Client Communication as a Driver of Burnout: A Scoping Review of Relational Risk and Protective Resources

**DOI:** 10.3390/vetsci13050411

**Published:** 2026-04-22

**Authors:** Mateus Eduardo Romão, Sara Rajae Beheshti, Simone Scoccianti, Serena Barello

**Affiliations:** 1 WHYpsy Lab, Department of Brain and Behavioral Sciences, University of Pavia, 27100 Pavia, Italy; sara.rajaebeheshti01@universitadipavia.it (S.R.B.); serena.barello@unipv.it (S.B.); 2 DVM, GPCert (PM&A), CVO Latuspet, Oxford OX2 9SX, UK; denebio@gmail.com; 3 Unit of Applied Psychology, IRCCS Mondino Foundation, 27100 Pavia, Italy

**Keywords:** burnout, veterinarians, communication, incivility, euthanasia, pet owners

## Abstract

Veterinary practice involves frequent interactions with pet owners that may be conflictual, emotionally charged, and ethically complex. These relational demands may contribute to burnout and related distress, but the evidence remains scattered across outcomes and study designs. This scoping review mapped the literature on how veterinarian–client communication and relational experiences are associated with burnout among veterinarians. Five databases were searched, and 17 studies met the inclusion criteria. Most were quantitative, with qualitative and mixed-methods studies also identified. Overall, the findings showed that repeated relational friction with clients, emotionally demanding end-of-life conversations, financial conflicts, and blurred boundaries around availability can contribute to burnout risk. The review also identified mechanisms linking these experiences to distress, particularly emotional reactivity and the effort required to regulate emotions during difficult interactions. Protective factors included supportive teams, autonomy, structured communication training, and interventions such as Acceptance and Commitment Training. These findings suggest that burnout in veterinary practice is shaped not only by workload, but also by the relational demands of client-facing care. Prevention efforts should therefore combine individual support with team-based and organizational strategies that strengthen communication practices and reduce avoidable interpersonal strain.

## Simple Summary

Veterinarians do much more than diagnosing and treating animals. They also have frequent and often emotionally difficult conversations with pet owners, especially when discussing serious illness, euthanasia, costs of care, or disagreements about treatment. These interactions can become a major source of stress and may contribute to burnout, emotional exhaustion, and other forms of distress. This study examined the existing research on how communication and relationships with clients affect veterinarians’ well-being. Seventeen studies were included. The findings showed that repeated conflict, rude behavior, unrealistic expectations, financial disputes, grief-related conversations, and pressure to remain constantly available can place a heavy emotional burden on veterinarians. At the same time, some factors appeared to protect well-being, including supportive colleagues, clear workplace policies, communication training, opportunities for debriefing, autonomy, and psychological skills that help professionals manage difficult emotions. Overall, the review suggests that burnout in veterinary practice is shaped not only by workload, but also by the relational demands of working with clients. Improving communication support, team practices, and organizational structures may help protect veterinarians’ mental health and support better care for animals and their owners.

## 1. Introduction

Veterinary practice is psychologically demanding, and chronic occupational stress may manifest as burnout and related negative outcomes (e.g., compassion fatigue, secondary traumatic stress disorder) [1,2,3]. Although workload, long working hours, and organizational pressures are frequently discussed as risk factors, an increasingly visible feature of contemporary veterinary work is that clinical care is delivered through sustained interaction with pet owners, often under conditions of uncertainty and constrained resources [4,5]. In this context, communication is not a neutral conduit for clinical information, but a core component of practicing veterinary medicine shaped by both the emotional texture of daily work and the interpersonal conditions under which ethically complex decisions are made.

Client-facing encounters can involve a distinctive and high emotional intensity, including end-of-life discussions, euthanasia decision-making, and grief support, but they may also include incivility, conflicts, and challenges to professional legitimacy. Across empirical studies, veterinarians describe situations in which they must navigate anger, accusations, distrust, and disrespect while maintaining composure and professionalism [6,7,8]. Such interactions appear consequential not only because they are unpleasant, but also because they may threaten professional identity by positioning veterinarians as uncaring or financially motivated and therefore undermining the relational foundation necessary for collaborative care [9,10]. Importantly, demands from clients may extend beyond acute conflict. Studies describe persistent “everyday” interaction stressors, such as excessive communications, non-adherence, and reliance on external advice, that require ongoing relational labor, accumulate over time, and may contribute to burnout risk in a manner comparable to more overt aggression [11,12].

The relational nature of veterinary practice also intersects with structural and moral constraints. Financial discussions are often identified as a key factor influencing veterinarian-client interactions, where constraints on owners’ ability or willingness to pay create tension, resentment, and moral stress about providing the “ideal” vs. “affordable” care [13]. In parallel, end-of-life care concentrates emotional labor and ethical strain. Veterinarians report difficulties in communication with bereaved clients, limited preparation for grief work, and distress associated with euthanasia dilemmas [14,15]. However, the existing evidence remains fragmented because these relational domains are typically examined in isolation and operationalized inconsistently across studies (e.g., using different outcomes, such as burnout, compassion fatigue, distress, etc.). The evidence is also marked by varied measures and definitions, heterogeneous designs, and, in some cases, mixed samples of veterinary roles. As a result, it is difficult to integrate findings and determine which client-related stressors are most consistently associated with burnout and which relational or organizational resources appear most protective across practice. These dynamics are not confined to any single practice setting; rather, they represent recurring relational domains through which veterinary work is experienced as emotionally burdensome, morally complex, and at times, socially contested.

Despite growing attention to veterinarians’ mental health, evidence on how veterinarian–client communication contributes to burnout remains fragmented across outcomes, relational domains, and study designs. This limits the development of targeted, practice-relevant interventions. The aim of this scoping review is therefore to synthesize and structure existing evidence on veterinarian–client communication as a source of occupational stress and burnout, identifying recurring relational demands, underlying mechanisms, and protective resources. Guided by the Job Demands–Resources model, the review provides an integrative framework to inform prevention strategies at individual, team, and organizational levels within veterinary practice.

## 2. Materials and Methods

### 2.1. Study Design

This scoping review followed JBI guidance and the PRISMA-ScR checklist. It was guided by the following research question: “What is known from the existing literature about how communication and relational experiences with clients contribute to burnout among veterinarians?”.

### 2.2. Eligibility Criteria

This review included primary, peer-reviewed studies involving veterinarians or veterinary professionals working in client-facing roles with pet owners, animal owners, or caregivers (e.g., small-animal practice, farm or large-animal practice, shelters, or emergency services). Eligible studies addressed burnout or related psychological outcomes, such as emotional exhaustion, compassion fatigue, or work-related distress, in relation to veterinarian-client communication or relational dynamics. Specifically, studies had to explicitly engage with relational or communicative domains, including (but not limited to) difficult conversations (e.g., euthanasia decision-making), client aggression, emotional labor, empathy, communication breakdowns, or conflict in care. Communication domains were operationalized as interpersonal exchanges or communicative processes occurring between veterinary professionals and clients, including information exchange, shared decision-making, explanation of diagnosis or treatment, end-of-life and euthanasia conversations, grief-related communication, cost-of-care discussions, conflict management, and others. Relational domains were operationalized as broader interpersonal dynamics characterizing the veterinary-client relationship, such as trust, incivility, aggression, blame, perceived respect to legitimacy, boundary intrusions, emotional labor arising from client interactions, and relationship strain or support. No geographical or practice-setting restrictions were applied. Studies with qualitative, quantitative, or mixed-methods designs were considered. Only studies published in English were considered. No secondary data, narrative reviews, editorials, dissertations, conference abstracts, or grey literature were included in this review.

### 2.3. Information Sources and Search

To identify suitable keywords, one researcher conducted an initial search on PubMed on 10 June 2025. Subsequently, two researchers collaborated to refine the search string for PubMed on 16 June 2025, which was adapted and used in other databases. The final search on PubMed was performed by one researcher (Table 1). The search was conducted in five databases: PubMed, Scopus, Web of Science, PsycINFO, and CINAHL.

### 2.4. Study Selection

**Figure 1:**
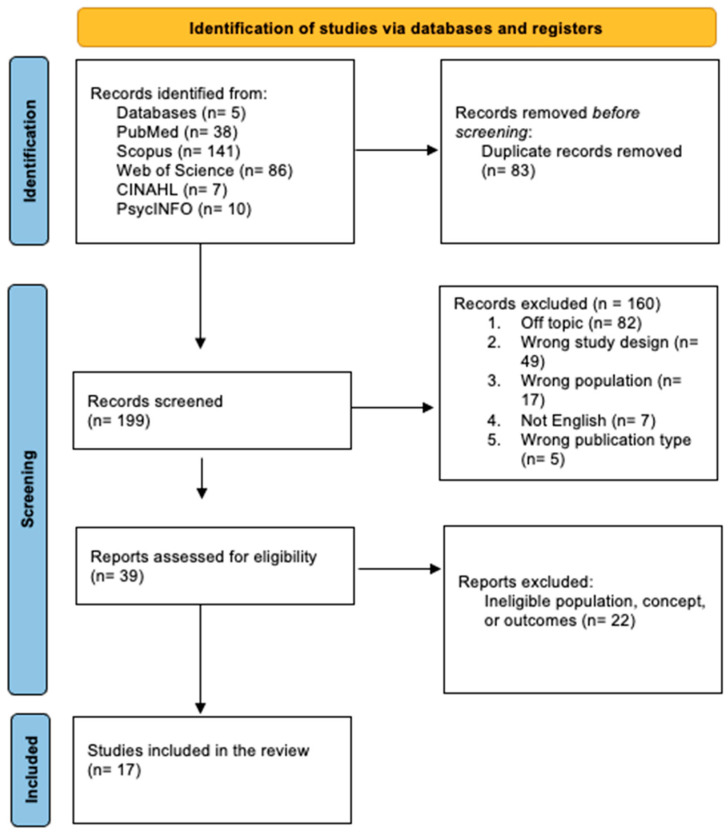
PRISMA flowchart of the search results.

Following the search string, as seen in Table 1, all identified references were downloaded in RIS or CSV format and uploaded to Rayyan (Rayyan Systems Inc., Cambridge, MA, USA) [16]. After removing the duplicates automatically, two researchers screened titles and abstracts. Potentially relevant studies were assessed for full text. During full-text screening, the reviewers applied the operational definitions of communication and relational domains reported in the eligibility criteria to determine whether client-facing interpersonal factors were an explicit analytic focus rather than a peripheral contextual detail. Any discrepancies between the reviewers were solved through peer discussion with a senior researcher. The search results and the studies included and excluded are comprehensively reported in the PRISMA-ScR extension flowchart, as seen in Figure 1 [17,18].

### 2.5. Data Extraction and Analysis

Full-text review and data extraction were conducted independently by two researchers. Two researchers developed extraction tables that contain information regarding the study’s characteristics and information to answer the research question of this scoping review, as seen, respectively, in Table 2 and Table 3.

### 2.6. Critical Appraisal

A critical appraisal of the included studies was conducted using the Mixed Methods Appraisal Tool (MMAT) [19]. The MMAT was selected because this review included studies of different study designs, and the tool allows appraisal across these designs within a single framework. Following MMAT guidance, each study was first assigned to the relevant study category and then assessed using the corresponding five methodological criteria. The two MMAT screening questions were considered prior to category-specific appraisal. In line with MMAT recommendations, no overall numerical score was calculated. Instead, the appraisal findings were reported criterion by criterion as “yes”, “no”, or “can’t tell”, and were used to contextualize the interpretation of the findings rather than to exclude studies. The full appraisal is presented in Supplementary Table S1A–D.

**Table 2:** Study characteristics.

Included Article	Country	Study Design	Population Characteristics	Burnout Measure	Psychological Outcomes
[6]	Canada	Qualitative	25 participants; 80% women; mean age 49.7; multiple practice types; included associates and owners.	N/A	Burnout, compassion fatigue/secondary traumatic stress, and distress
[7]	UK	Qualitative	18 veterinarians; 16 women, 2 men; mean age 35; small-, mixed-, and farm-practice settings.	N/A	Emotional exhaustion and distress
[8]	USA	Qualitative	124 veterinarians; 75.8% female; mean age 49.4; mainly private practice.	N/A	Distress
[12]	USA	Quantitative	1151 small-animal veterinarians; 81% female; mean age 46.	Copenhagen Burnout Inventory (CBI)	Burnout
[14]	Australia	Quantitative	105 veterinary surgeons; 63.1% female; mean age 33.5; mainly companion, equine, or mixed practice.	Compassion Fatigue Short Scale (CFSS).	Compassion fatigue/secondary traumatic stress and distress
[20]	Australia	Mixed methods	249 participants; mainly clinical staff (67.1%); specialist small-animal hospital; mostly female (69.9%).	Maslach Burnout Inventory—General Survey (MBI-GS).	Burnout and distress
[21]	Australia & New Zealand	Qualitative	53 veterinary professionals, including veterinarians, nurses, and technicians.	N/A	Burnout, distress and moral distress
[22]	USA	Quantitative	34 participants; mostly female (94.1%); mean age 35; all in small-animal practice.	Copenhagen Burnout Inventory (CBI).	Burnout
[23]	USA	Quantitative	137 veterinary team members; 94.9% female; mean age 38; mixed client-facing roles across referral, urgent, emergency, and general practice.	Copenhagen Burnout Inventory (CBI).	Burnout and distress
[24]	Austria	Qualitative	20 small-animal veterinarians; 18 women, 2 men; mostly self-employed; from Germany, Switzerland, and Austria.	N/A	Burnout, emotional exhaustion, and distress
[25]	Italy	Quantitative	704 veterinarians; companion, farm, or both; 70.3% women.	Maslach Burnout Inventory (MBI).	Burnout (depersonalization and cynicism), distress and moral distress
[26]	Canada	Mixed-methods	Mixed-methods sample: 20 interview participants + 990 survey respondents; veterinarians and animal health technologists across small-, mixed-, and large-animal settings.	Professional Quality of Life (ProQOL).	Compassion fatigue/secondary traumatic stress
[27]	USA	Qualitative	86 veterinary professionals; 92% female; ages 25–65+; mainly mobile or clinic-based practice.	N/A	Burnout, compassion fatigue/secondary stress and distress
[28]	USA	Quantitative	1122 small-animal veterinarians (US/Canada); 68.5% female; owners and associate/relief veterinarians; median 15 years in practice.	N/A	Burnout, distress, moral distress, and turnover intention
[29]	Australia	Quantitative	136 veterinary professionals (43 veteri-narians, 93 nurses); 94.9% female; mainly private clinics and some shelters.	Professional Quality of Life (ProQOL).	Burnout, com-passion fa-tigue/secondary traumatic stress and turnover intention
[30]	USA	Quantitative	232 veterinary personnel, including associate veterinarians and practice owners/partners.	Professional Quality of Life (ProQOL).	Compassion fatigue and secondary traumatic stress
[31]	Portugal	Quantitative	229 veterinarians + 96 veterinary nurses; mostly female; mean ages 34.5 and 28.5; mainly private sector.	Oldenbug Burnout Inventory (OLBI).	Burnout and emotional exhaustion

**Table 3:** Extraction table from the included studies.

Included Article	Communication/Relational Variables	Job Demands	Job Resources	Personal Resources	Qualitative Key Themes	Implications for Practice
[6]	Distrust, financial conflict, aggression, lack of appreciation; pets as family increased pressure	Emotional load, unrealistic expectations, moral stress, aggression, heavy caseloads	Team support and mentorship	Self-compassion, perspective-taking, separating own vs. client values	Professional demands; relationships; financial strain; moral distress	Conflict/business training, mentorship, protected time
[7]	Incivility, yelling, accusations of being uncaring or money-driven; coping through empathy and assertiveness	Client rudeness, financial conflict, pressure to stay professional	Collegial support; practical client-management strategies	Self-care, reframing, empathy, proactive planning	Proactive client management	Boundary setting, co-consultation, and immediate debriefing
[8]	“Double-faced” emotion management: suppressing emotion, validating clients, using logic/facts	Unrealistic demands, disrespect, powerlessness, aggression/cyberbullying, death work	Backstage support; limited training noted	Self-care, reframing, coping backstage, sometimes maladaptive coping	Feeling overwhelmed, frustrated, fearful, powerless while staying “strong”	Communication and emotional labor training; realistic job preparation
[12]	Communication; response to clients predicted stress/burnout more than exposure frequency	Burden transfer, grief/euthanasia strain, nonpayment, unrealistic expectations	Need for skills-based training and structured support	Coping, emotional regulation, reactivity management	N/A	Reduce caregiver burden and teach response skills; BTI useful
[14]	Difficulty communicating with grieving clients; felt underprepared	Client grief, euthanasia distress, ethical dilemmas, long hours	Debriefing with colleagues; limited communication/social support training	Detachment; older age	N/A	Grief communication training; compassion fatigue support; early-career programs
[20]	Managing client emotions, aggression, difficult financial discussions, lack of respect	Heavy workload, long hours, low recognition, emotional and ethical strain	Team cohesion, support, meaning, autonomy, learning opportunities	Meaning, self-efficacy, pride, emotional regulation	Stressors: communication, clients, caseload; Well-being: people, practice, purpose	Schedule autonomy, support culture, emotional regulation training
[21]	Challenging clients, blame, refusal to pay, unrealistic expectations; positive clients could also protect	Difficult clients, emotional blackmail, financial strain, long hours, understaffing	Team cohesion, gratitude, good work conditions, positive client relationships	Healthy habits, trusted relationships	Negative work conditions; challenging relationships; limited support	Organizational responsibility; improve staffing and flexibility
[22]	DANCE model: daily hassles, affective strain, non-adherence, confrontation, excessive communication; reactivity mattered more than frequency	Grief/euthanasia counseling, complaints/blame, unrealistic expectations, non-adherence, excess contact	Group ACT	Psychological flexibility, values-based coping	N/A	ACT acceptable and feasible; larger trials needed
[23]	ACT intervention targeting reactivity to difficult client interactions	DANCE-related burden transfer and stressors	ACT in live and self-paced formats	Acceptance, resilience, defusion, values-based action	N/A	Self-paced training can reduce client-related burnout
[24]	High client expectations, poor respect for boundaries, grief, ICT contact, overpersonalization	24/7 availability, after-hours calls/messages, emergencies, emotional spillover, role conflict	Autonomy, formal boundaries, clinic as safer space	Self-care mindset, emotional regulation, ICT management	Work–private blur; boundary setting; self-care after negative experiences	ICT boundary training, formal communication rules, self-care orientation
[25]	Conflict with owners/farmers, colleague disagreement, poor communication, euthanasia pressure	Client conflict, refusal to pay, role conflict, workload, legal/institutional stress	N/A	Professional experience	N/A	Ethics/communication training; moral stress support
[26]	Positive bonds with animals/clients but also barriers, unrealistic expectations, grief	Financial constraints, communication difficulties, grief, euthanasia, complex cases	Positive client relationships, helping animals, animal bonds	N/A	Paradox of compassionate work: same bonds can satisfy and cause fatigue	Use multilevel interventions; distinguish satisfaction from fatigue
[27]	“Dr. Google,” expectation management, delayed help-seeking, difficult financial discussions	Emotional load, misinformation correction, emergencies, workload	Client gratitude, clear fees, home visits	Reframing, meaning-making, impression management, coping	Internet information and EOL knowledge gaps	Use websites, early intervention, communication workshops
[28]	Cost discussions, client economic limits, resentment, “mercenary” accusations	Economic constraints, fee disputes, moral stress, time pressure, economic euthanasia	Credit, payment plans, financial aid, insurance	Experience, autonomy, client education	N/A	Early cost communication, insurance promotion, longer appointments
[29]	Emotionally charged client interactions, daily hassles, indirect euthanasia exposure	Emotional load, role conflict, daily hassles	Social support, feedback, development opportunities, autonomy	Optimism, positive reframing, less disengagement	N/A	Support optimism, autonomy, and development
[30]	Empathy, perspective-taking, toxic communication	Toxic team climate, emotional distress, traumatic exposure	Team engagement, coordinated environment, cognitive empathy	Perspective-taking, engagement, age/tenure	N/A	Improve culture, recognition, and trauma-informed debriefing
[31]	Human-directed empathy (affective and cognitive)	Emotional overload, workload, euthanasia-related stress	Perceived justice, professional identity, meaningful work	Empathy	N/A	Strengthen cognitive empathy, regulate affective empathy

## 3. Results

### 3.1. Search Results

Initially, 282 records were identified. After the automatic removal of 83 duplicates, 199 studies were screened. Of these, 160 were excluded for not meeting the inclusion criteria. Thirty-nine full-text articles were assessed for eligibility, and 17 studies met all inclusion criteria and were included in this scoping review. The screening process is detailed in Figure 1.

### 3.2. Study’s Characteristics

Seventeen studies were included in the final version of this scoping review. The majority of studies were conducted in the United States (*n* = 7, 41%), followed by Australia (*n* = 3, 17%) and Canada (*n* = 2, 11%); one study each came from Portugal, the United Kingdom, Italy, Austria, and Australia/New Zealand. Most of the studies adopted a quantitative design (*n* = 9, 52.9%), followed by qualitative (*n* = 6, 35.3%), and mixed methods (*n* = 2, 11.8%). Across studies, a total of 5.531 veterinary professionals were represented, including veterinarians or veterinary team members. Standardized burnout or related instruments were reported in 10 studies, the most commonly used were the Copenhagen Burnout Inventory (CBI) and the Professional Quality of Life Scale (ProQOL), and 7 studies did not report a standardized burnout measure. For a detailed overview of study characteristics, see Table 2.

### 3.3. Critical Appraisal of Included Studies

The MMAT appraisal indicated that the included studies were methodologically heterogeneous. Overall, the evidence base showed variable rigor, with clearer strengths and more frequent limitations in the quantitative descriptive literature. Common strengths included the use of appropriate qualitative approaches, validated psychometric scales, and, in some cases, explicit integration of qualitative and quantitative components. Common limitations included cross-sectional designs, voluntary or convenience sampling, low or unclear response rates, and limited representativeness, which reduce confidence in causal inference and generalizability. Among the randomized studies, intervention designs and standardized outcome measures were strengths, but blinding was not evident, and outcome completeness was limited in the larger trial. Among the qualitative studies, interview-based papers generally showed stronger coherence between research question, data collection, and analysis. In contrast, studies based on open-ended survey responses were informative but offered less interpretative depth. In mixed-methods studies, the contribution was strongest when the qualitative and quantitative strands were clearly connected in design and interpretation. The full criterion-level appraisal is reported in the Supplementary Table S1A–D. 

### 3.4. Main Findings

Six main themes were developed from the extracted findings of the included studies using Braun and Clarke’s (2006) [32] thematic analysis framework, which supports the systematic identification and reporting of patterns (or themes) across a dataset. Guided by the Job Demands–Resources (JD-R) model [33], the themes were interpreted as capturing client-related job demands (e.g., emotionally demanding conversations, conflict, incivility, financial disputes, and others), job resources (e.g., team cohesion, debriefing, mentorship, autonomy, and structured training), and personal resources (e.g., emotion regulation strategies, psychological flexibility, self-compassion, and regulated empathy). Beyond identifying recurring client-related stressors, the qualitative evidence suggested that these demands were often experienced as emotionally cumulative and difficult to contain, especially when veterinarians felt expected to remain composed in highly charged interactions. Moreover, Table 3 presents the findings retrieved from the original articles.

#### 3.4.1. Relational Friction with Clients Becomes a Chronic Emotional Demand

Across studies, veterinarian–client communication emerged as a chronic source of emotional demand, in which repeated relational friction accumulated over time and contributed to the risk of burnout. Studies described the client as at times disrespectful or hostile (e.g., incivility, yelling, blame, or accusations of being uncaring), with these encounters experienced as identity-threatening and difficult to metabolize emotionally while maintaining professionalism [6,7,8,20,21]. This relational strain was also intensified by a mismatch of expectations, including perceptions of pets as family members, which amplified emotional intensity and increased the pressure placed on veterinarians to satisfy client demands under conditions of uncertainty, limited resources, or clinical constraints [6,20,21]. In parallel, multiple studies highlighted everyday “hassle” type of stressors, such as excessive communications, non-adherence or inconsiderate behaviors, and reliance on external advice, which contributed to ongoing friction and to a chronic sense of being gradually diminished by encounters with clients [12,22,23].

#### 3.4.2. Reactivity and Emotional Regulation as a Mechanism Linking Client Stressors to Burnout Risk

Reactivity to difficult client interactions emerged as a central mechanism linking client-related demands to burnout risk. Specifically, within the DANCE/burden transfer framing, veterinarians’ internalisation of and emotional reactions to challenging client behaviors appeared more consequential than the frequency of those behaviors, indicating that burnout risk is shaped by the interaction between relational demands and clinicians’ psychological responses [12,22]. Qualitative studies suggested that client-related stress became harmful not simply because interactions were difficult, but because veterinarians often had to remain composed while absorbing clients’ anger, grief, or distrust. Participants described feeling overwhelmed, frustrated, powerless, or fearful, yet still needing to appear neutral, logical, and reassuring. Emotional self-management, therefore, emerged not as a secondary issue, but as part of the work itself [7,8,14]. Other studies reported detachment and emotional distancing as a pragmatic coping strategy, particularly in the face of death and boundary management, implying that emotional self-protection may be simultaneously adaptive and indicative of the emotional costs embedded in relational labor [14,24]. Collectively, these findings position client-facing veterinary practice as a setting where sustained emotion regulation and reactivity management function as a core personal resource.

#### 3.4.3. End-of-Life and Death Communication Concentrates Moral Distress and Emotional Labor

Euthanasia and bereavement-related conversations were consistently described as high-intensity interactions that concentrate emotional labor and moral distress. Across studies, veterinarians reported difficulty communicating with grieving clients and perceived limited preparation for grief work, commonly relying on informal debriefing and emotional distancing as coping strategies [14]. Euthanasia further appeared as a complex relational event involving ethical dilemmas, including distress linked to “convenience euthanasia” and disagreements or pressure surrounding treatment decisions, which contributed to strained client relationships and emotional communication overload [12,25]. This relational burden was not uniformly negative. Some studies highlighted that meaningful bonds with clients and their pets could work as a protective factor against burnout. However, the same relational closeness was also seen as a way to fatigue, supporting the paradox in which compassionate connection is a complex phenomenon [21,26].

#### 3.4.4. Economic Constraints Turn Communication into Conflict and Moral Strain

Financial constraints emerged as a prominent relational determinant of burnout risk by repeatedly converting care discussions into interpersonal conflict and moral stress, rather than functioning solely as a logistical barrier. Across studies, cost-of-care conversations were described as challenging due to barriers to discussing fees, clients’ economic limitations, aggressive refusal to pay or disputes over value for money, and resentment that accumulated over time [7,21,27,28]. These exchanges often carried a strong moral and identity component: veterinarians reported being accused of acting in a mercenary way or being financially motivated, which undermined relational trust and placed clinicians in a defensive communicative position [7,8,28]. Economic constraints also shaped clinical trajectories, including the modification of advice based on prior interactions and the burden of deciding financial limitations, sometimes intersecting with euthanasia-related dilemmas and moral distress [25,28].

#### 3.4.5. Erosion of Boundaries and Authority in the Always-Connected, Information Saturated Client Context

Boundary erosion and challenges to professional authority were less frequently examined, but emerged as salient stressors in the studies that addressed them. For example, one qualitative study highlighted constant availability expectations and after-hours contact via calls or messages, which blurred work–private boundaries, reduced recovery time, and increased emotional load through persistent accessibility [24]. In the qualitative literature, this was experienced less as a simple scheduling problem and more as a lived intrusion of work into private life, making it harder to switch off, protect personal time, and maintain distance from clients’ situations [24,27]. In parallel, clients’ reliance on self-directed online information or advice was seen by the veterinarians as creating additional corrective communication labor, needing to address misinformation, manage expectations about the dying process, and handle delayed help-seeking behavior that could escalate clinical complexity [27]. Within this context, communication work expands beyond the clinic encounter into ongoing expectation management and relationship maintenance, intensifying demands even in the absence of overt conflict [24,27].

#### 3.4.6. Buffers and Interventions Operate Across Individual, Team, and Organizational Levels

Buffers were most consistently described as multi-level resources that either reduce client-related relational demands or strengthen clinicians’ capacity to manage them. In particular, Acceptance and Commitment Training (ACT) interventions were presented as relevant to client-related burnout because they directly targeted reactivity and burden transfer, thereby supporting psychological flexibility and values-based coping; self-paced formats were described as feasible and effective within busy clinical schedules [22,23]. Beyond individual approaches, team and organizational resources repeatedly appeared as protective: collegial support, debriefing opportunities, feedback, autonomy, team cohesion, and professional development were identified as buffers against emotionally charged client interactions and euthanasia-related stress [7,14,20,21,29]. Studies also emphasized training gaps and the need for structured preparation in grief communication, emotional labor, empathy regulation, and conflict management, alongside mentorship, especially in younger professionals [6,8,14,25,28]. Finally, individual personal resources, including self-compassion, optimism/positive reframing, perceived justice, and others, were described as shaping vulnerability or resilience, with evidence that empathy is not uniformly protective and may require regulation, such as affective vs. cognitive empathy distinctions [29,30,31]. 

## 4. Discussion

This scoping review synthesised the literature on how veterinarian–client communication and relational experiences may contribute to burnout and related distress. Overall, the findings indicate that client-facing work is characterised by recurring interpersonal and ethical challenges, such as incivility, emotionally demanding encounters, financial disputes, and end-of-life conversations, occurring alongside variable access to protective resources (e.g., collegial support, structured training, autonomy, psychological flexibility, and self-compassion) [33]. Collectively, the evidence suggests that veterinarian–client relational experiences are not peripheral stressors, but part of the routine working conditions through which strain can accumulate, and well-being may be protected or undermined.

Across studies, repeated relational friction with clients emerged as a chronic emotional demand. Incivility, accusations, hostility, and distrust were described as particularly burdensome when they challenged veterinarians’ professional identity and required maintaining composure while being positioned as uncaring or financially motivated [6,8,11,20]. Comparative evidence further suggests that reputational vulnerability and complaint infrastructures can amplify these pressures, particularly when conflicts become public and persistent [34]. Taken together, these findings indicate that conflict is not only a matter of “difficult clients,” but may reflect broader contextual drivers that influence expectations, trust, and escalation pathways.

A second consistent pattern was the centrality of reactivity and emotion regulation in linking client-related demands to burnout risk. Work using the burden transfer/DANCE framing suggests that clinicians’ responses to difficult client behaviors may be as consequential as how often those behaviors occur [12,22,23]. Qualitative evidence similarly described the need to suppress negative emotions, validate clients, and avoid escalation as an effortful form of professional performance [8], with detachment and emotional distancing reported as pragmatic coping strategies in high-intensity situations [14,24]. Taken together, these findings suggest that burnout risk is shaped not only by exposure to difficult client encounters but by the repeated need to contain their emotional impact while continuing to appear calm, credible, and professionally available. In this sense, emotional self-management appears to function as a hidden but central component of client-facing veterinary work. Importantly, these findings support the relevance of interventions that explicitly target reactivity and psychological flexibility, including Acceptance and Commitment Training, which was reported as feasible and beneficial even in self-paced formats [22,23]. This aligns with broader synthesis work that highlights client interactions as a major driver of stress and calls for actionable mitigation strategies [35,36].

End-of-life communication, particularly around euthanasia, was repeatedly described as a concentrated source of emotional labour and moral strain. Veterinarians reported difficulty communicating with bereaved clients, limited preparation for grief work, and distress linked to ethically complex decisions [14,25]. Evidence from Deponti et al. [37] reinforces that euthanasia-related distress is also a preparedness issue, with many veterinarians reporting gaps in training related to animal death, euthanasia/dysthanasia, mental health, and communication skills, alongside guilt and sadness following euthanasia. The implication is practical: where exposure to death-related encounters is inevitable, the modifiable variable is the availability of training, supervision, and structured support that enable clinicians to engage in these conversations without carrying the burden alone.

Financial constraints and cost-of-care discussions were consistently framed as relationally challenging, often converting clinical encounters into conflicts over value, responsibility, and professional legitimacy [7,8,21,28]. These conversations were described as demanding not only because they involve money, but because they can provoke moral stress (ideal vs. affordable care) and defensive communication when veterinarians feel accused of financial motivation. At the same time, communication may also act as a protective resource when it supports trust, shared understanding, and continuity. Consistent with this, owner-rated shared decision-making was positively associated with consultation satisfaction [35], and pet-owner loyalty research suggests that communication quality contributes to trust and commitment intentions [38]. While these outcomes are not direct measures of clinician well-being, they plausibly indicate relational conditions that may reduce downstream conflict and, consequently, lessen cumulative strain.

Although addressed in fewer studies, boundary erosion linked to information and communication technologies and heightened availability expectations appeared as a meaningful demand. After-hours contact, ongoing expectation management, and addressing client misinformation extended client-facing work beyond scheduled consultations and reduced opportunities for detachment and recovery [24,27]. This suggests that burnout prevention should also consider clinic-level norms and policies regarding after-hours communication, rather than treating availability as an implicit expectation borne by individual clinicians.

### 4.1. Implications for Veterinary Practice

The findings support multilevel actions that can be operationalized more concretely in routine veterinary practice. At the organizational level, clinics and hospitals may benefit from structured approaches to recurrent high-strain situations, particularly end-of-life discussions, cost-of-care conversations, and client incivility. These may include agreed communication frameworks, written fee and follow-up policies, escalation pathways for aggressive client encounters, and clearer norms regarding after-hours contact. Because workload was repeatedly identified as a key stressor, organizational responses should also extend beyond simply asking clinicians to “cope better.” More feasible strategies may include protected catch-up time, fuller use of veterinary technicians’ skill sets, greater role clarity, and, where possible, some degree of autonomy or choice over schedules and shifts. Evidence from specialist hospital settings suggests that autonomy and control over work time may reduce the emotional impact of heavy workload more effectively than workload reduction alone [6,20].

At the team level, the review suggests that support structures are likely to be most useful when they are specific, routine, and psychologically safe. Debriefing after euthanasia, hostile client interactions, or morally difficult cases should not be left entirely informal. Brief, structured case reflection may be more effective when it examines not only emotional reactions, but also interactional triggers, system contributors, and concrete changes for future encounters. Studies in the review also suggest that conflict within teams, unclear responsibilities, and lack of appreciation can themselves become stress amplifiers [6,20]. Accordingly, interventions may need to include explicit conflict-management procedures, clearer delineation of job roles, and deliberate supervisory practices that reinforce appreciation, trust, and permission to seek help without reputational risk.

At the training level, the evidence supports moving beyond general calls for “more communication training” toward more targeted content. Training should prepare veterinarians and other client-facing staff for emotionally demanding client encounters by including skills in grief communication, cost-of-care discussions, conflict de-escalation, emotional self-regulation, and management of residual distress after difficult consultations. The ACT-based interventions included in this review are especially relevant because they target reactivity to difficult client encounters rather than assuming that the client interaction itself can be eliminated. Importantly, the evidence also suggests that delivery format matters. Self-paced training may improve completion and scalability in busy settings, whereas live formats may feel more engaging or supportive. A practical implication is that blended or modular formats may be preferable, especially when paired with brief booster sessions over time [8,12]. Another underexplored but potentially important high-stakes communication context concerns the recognition and reporting of suspected animal cruelty, neglect, or linked household abuse. Although this issue was not directly examined across the included studies, it plausibly intersects with the relational pressures identified in this review by increasing the risk of distrust, defensive communication, hostility, and moral stress. In practice, this reinforces the importance of clear clinic policies, training on recognition and reporting procedures, and opportunities for team discussion or debriefing around such cases [39,40].

Implementation barriers should also be acknowledged explicitly. Time pressure, unpredictable clinical demands, and feeling too busy or overwhelmed may reduce uptake even when interventions are acceptable and evidence-informed. Mentorship and training programs may also be difficult to sustain when experienced staff lack time to support junior colleagues meaningfully. At the organizational level, some interventions may require upfront investment, such as protected training time, hiring a practice manager, or redesigning workflow. In addition, emotionally demanding aspects of client-facing care cannot be removed entirely, so interventions should be designed not only to reduce exposure where possible but also to improve staff preparedness and recovery [6,8,12]. For this reason, implementation is likely to be most realistic when interventions are embedded into routine systems, such as onboarding, continuing education platforms, supervision structures, and clinic communication policies, rather than offered as one-off wellness initiatives.

Overall, the evidence suggests that the most promising approach is not a single intervention, but a coordinated package combining organizational supports, team-based practices, and targeted training. This is especially important because the relational burdens identified in this review arise from ordinary features of veterinary work rather than isolated exceptional events. As a result, interventions are more likely to succeed when they are implemented as part of everyday professional infrastructure rather than as optional add-ons.

### 4.2. Limitations and Future Directions

Several limitations should be acknowledged. The MMAT appraisal indicated that much of the evidence base was limited by cross-sectional designs, voluntary or convenience-based sampling, and variable reporting quality, all of which should be considered when interpreting the consistency, transferability, and generalizability of the findings. First, as expected in a scoping review, the included evidence was heterogeneous in study designs, settings, and outcome operationalization, with a non-trivial proportion of studies not using standardized burnout psychometric instruments, which limits comparability across studies and precludes any quantitative estimation of effects. In addition, the evidence base was geographically and contextually concentrated, with most studies conducted in Western countries and in small-animal or companion-animal settings. This limits the generalizability of the findings to other veterinary contexts, including large-animal, mixed, farm-animal, equine, shelter, emergency, and non-Western practice environments, where client relationships, economic pressures, organizational structures, and end-of-life decision-making may take different forms. Second, most of the quantitative studies were cross-sectional, and the qualitative evidence, while highly informative for understanding perceived relational demands and coping processes, cannot establish temporal or causal pathways. Accordingly, the current evidence does not allow firm conclusions about whether client-related communication stressors contribute to burnout over time, whether veterinarians already experiencing burnout perceive and react to client interactions as more stressful, or whether these associations are bidirectional. The temporal sequencing between client-related demands, reactivity, emotion regulation, resource availability, and burnout therefore remains insufficiently tested. Future research should prioritize longitudinal, prospective, and diary/experience-sampling designs to clarify temporal ordering and test whether repeated client-related demands, emotional reactivity, and limited relational or organizational resources predict subsequent burnout over time. It should also more deliberately target underrepresented veterinary contexts, including large-animal, mixed, equine, shelter, emergency, and non-Western settings, to determine whether the relational stressors and protective resources identified in this review operate similarly across diverse professional environments. Future research should also examine high-stakes reporting contexts, such as suspected animal cruelty, neglect, or household abuse linked to abuse, as these situations may represent an important yet underexplored source of communication strain, moral stress, and burnout risk in veterinary practice. It should also employ standardized and well-validated measures of burnout and evaluate multilevel interventions that combine organizational policies (e.g., boundary management, structured approaches to cost-of-care discussions, and anti-abuse protocols) with team-based resources and individual skills training.

## 5. Conclusions

This review indicates that burnout risk in veterinary practice is not only a consequence of workload, but also of relational exposure, in which repeated client-facing interactions can operate as high-intensity job demands (e.g., conflict, grief, financial disputes, and boundary intrusions) that accumulate when recovery and organizational supports are insufficient. Addressing burnout in veterinary medicine therefore requires system-level responses that recognize veterinarian–client communication as a core occupational demand and invest in relational, organizational, and educational resources to support sustainable practice.

## Figures and Tables

**Table 1:** PubMed search string.

Database	Search String
PubMed	(“Veterinarians”[MeSH] OR veterinarian*[tiab] OR “veterinary professional*”[tiab] OR “veterinary surgeon*”[tiab]) AND (“Burnout, Professional”[MeSH] OR “Compassion Fatigue”[MeSH] OR “Stress, Psychological”[MeSH] OR burnout[tiab] OR “emotional exhaustion”[tiab] OR “compassion fatigue”[tiab] OR “occupational stress”[tiab]) AND (“Professional-Patient Relations”[MeSH] OR “Communication”[MeSH] OR “Interpersonal Relations”[MeSH] OR “Client interaction”[tiab] OR “pet owner*”[tiab] OR “animal owner*”[tiab] OR “difficult conversation*”[tiab] OR “client aggression”[tiab] OR “empathy”[tiab] OR “relationship with client”[tiab])

MeSH = Medical Subject Headings; tiab = title/abstract; * = truncation symbol used to retrieve multiple word endings.

## Data Availability

No new data were created or analyzed in this study. Data sharing is not applicable to this article.

## Supplementary Materials

The following supporting information can be downloaded at: https://www.mdpi.com/article/10.3390/vetsci13050411/s1, Table S1A: MMAT appraisal of qualitative studies; Table S1B: MMAT appraisal of randomized controlled trials; Table S1C: MMAT appraisal of quantitative descriptive studies; Table S1D: MMAT appraisal of mixed-methods studies.
