# Supplementary material for: Veterinarian–Client Communication as a Driver of Burnout: A Scoping Review of Relational Risk and Protective Resources
Source: Vet Sci. 2026 Apr 22;13(5):411. doi: 10.3390/vetsci13050411 (PMC13211551; doi:10.3390/vetsci13050411)
Supplement: Supplementary file 1 [file vetsci-13-00411-s001.zip › vetsci-4250092-supplementary.pdf]

Supplementary Table S1A. MMAT appraisal of qualitative studies

Criteria
1.1 Is the qualitative approach appropriate to answer the research question?
1.2 Are the qualitative data collection methods adequate to address the research question?
1.3 Are the findings adequately derived from the data?
1.4 Is the interpretation of results sufficiently substantiated by data?
1.5 Is there coherence between qualitative data sources, collection, analysis and interpretation?

Study	1.1	1.2	1.3	1.4	1.5	Brief appraisal note
Romo et al. (2025)	Y	CT	Y	Y	Y	Clear qualitative focus and coherent analysis, but the use of an open-ended online questionnaire reduced depth compared with interview-based designs.
Irwin et al. (2022)	Y	Y	Y	Y	Y	Strong fit between question, interviews, and thematic analysis; self-selected sample limits transferability.
Connolly et al. (2022)	Y	CT	Y	Y	Y	Reflexive thematic analysis was appropriate, but the data were generated through only two open-ended survey questions and had limited contextual depth.
Campbell et al. (2025)	Y	Y	Y	Y	Y	One of the strongest qualitative studies; in-depth interviews, explicit analytic approach, and attention to saturation and rigour.
Dürnberger & Springer (2025)	Y	Y	Y	Y	Y	Detailed semistructured interviews, transparent coding process, and explicit discussion of bias and reflexivity.
Hoffmann & Dickinson (2021)	Y	CT	Y	Y	CT	Relevant exploratory qualitative analysis, but open-ended survey responses and uncertain response rate reduced interpretive depth and contextual coherence.

Supplementary Table S1B. MMAT appraisal of randomized controlled trials

Criteria
2.1 Is randomization appropriately performed?
2.2 Are the groups comparable at baseline?
2.3 Are there complete outcome data?
2.4 Are outcome assessors blinded to the intervention provided?
2.5 Did the participants adhere to the assigned intervention?

Study	2.1	2.2	2.3	2.4	2.5	Brief appraisal note
Spitznagel et al. (2021)	Y	CT	CT	N	Y	Appropriate pilot randomized design with standardized measures, but limited reporting on baseline comparability, outcome completeness, and blinding.
Spitznagel et al. (2023)	Y	CT	N	N	CT	Clear randomized comparison with repeated follow-up, but substantial attrition from randomized enrollment to completed assessments and no clear blinding.

Supplementary Table S1C. MMAT appraisal of quantitative descriptive studies

Criteria
4.1 Is the sampling strategy relevant to address the research question?
4.2 Is the sample representative of the target population?
4.3 Are the measurements appropriate?
4.4 Is the risk of nonresponse bias low?
4.5 Is the statistical analysis appropriate to answer the research question?

Study	4.1	4.2	4.3	4.4	4.5	Brief appraisal note
Varela & Correia (2023)	Y	N	Y	CT	Y	Appropriate measures and analyses, but online recruitment via Facebook/LinkedIn produced a convenience sample with uncertain representativeness.
Spitznagel et al. (2019)	Y	N	Y	N	Y	Strong psychometric development and validation procedures, but the veterinarian sample was self-selected and nonresponse bias is likely.
Dow et al. (2019)	Y	N	Y	N	Y	Relevant sampling frame and standardized instruments, but low/uncertain response rate and cross-sectional self-report limit confidence.
Rohlf et al. (2022)	Y	N	Y	CT	Y	Useful predictor analysis with established measures, but representativeness was limited and nonresponse bias was not clearly resolved.
Pizzolon et al. (2019)	Y	N	Y	CT	Y	Established instruments and appropriate modeling were strengths, but clinic selection and response structure limit representativeness.
Prato-Previde et al. (2024)	Y	CT	Y	CT	Y	Large national voluntary sample, explicit factor analysis, and strong reliability reporting; representativeness remains uncertain because recruitment was online and voluntary.
Kipperman et al. (2017)	Y	N	CT	N	Y	Large sample and clinically important topic, but low response fraction and reliance on partly custom survey items weaken methodological confidence.

Supplementary Table S1D. MMAT appraisal of mixed-methods studies

Criteria
5.1 Is there an adequate rationale for using a mixed-methods design to address the research question?
5.2 Are the different components effectively integrated to answer the research question?
5.3 Are the outputs of the integration of qualitative and quantitative components adequately interpreted?
5.4 Are divergences and inconsistencies between quantitative and qualitative results adequately addressed?
5.5 Do the different components adhere to the quality criteria of each tradition of the methods involved?

Study	5.1	5.2	5.3	5.4	5.5	Brief appraisal note
Polachek & Wallace (2018)	Y	Y	Y	CT	CT	Clear sequential mixed-methods rationale and useful integration, but divergences were not deeply explored and the component quality was uneven.
Ashton-James & McNeilage (2022)	Y	Y	Y	CT	Y	Stronger mixed-methods study with explicit convergence between survey and focus-group findings, though inconsistencies were not a major analytic focus.
